# *Bacillus thuringiensis* Suppresses the Humoral Immune System to Overcome Defense Mechanism of *Plutella xylostella*

**DOI:** 10.3389/fphys.2018.01478

**Published:** 2018-11-15

**Authors:** Shuzhong Li, Xiaoxia Xu, Muhammad Shakeel, Jin Xu, Zhihua Zheng, Jinlong Zheng, Xiaoqiang Yu, Qian Zhao, Fengliang Jin

**Affiliations:** ^1^Key Laboratory of Bio-Pesticide Innovation and Application of Guangdong Province, College of Agriculture, South China Agricultural University, Guangzhou, China; ^2^Institute of Insect Science and Technology, School of Life Sciences, South China Normal University, Guangzhou, China; ^3^Beijing Genomics Institute, Shenzhen, China

**Keywords:** *Plutella xylostella*, *Bacillus thuringiensis*, insect immunity, transcriptome, digital gene expression profiling, antimicrobial peptides

## Abstract

**Background:** *Plutella xylostella* has become a notorious pest of cruciferous crops all over the world. Delta-endotoxins of *Bacillus thuringiensis* are widely used insecticidal proteins for controlling *P. xylostella*. However, the interaction mechanism of *B. thuringiensis* with the immune system of *P. xylostella*, at the genomic level, is still unclear. This study explored the immune response of *P. xylostella* to *B. thuringiensis*, at different time intervals, 6 h, 12 h, 18 h, 24 h, and 36 h, by using RNA-Sequencing (RNA-Seq) and RT-qPCR.

**Results:** In total, 167 immunity-related genes were identified and placed into different families, including pattern recognition receptors (PRRs), signal modulators, immune pathways (Toll, IMD, and JAK/STAT), and immune effectors. It is worth mentioning that the analyses of the differentially expressed immunity-related genes revealed that most of the differentially expressed genes (DEGs) (87, 56, 76, 67, and 73 genes) were downregulated in *P. xylostella* following *B. thuringiensis* oral infection at 6 h, 12 h, 18 h, 24 h, and 36 h. Interestingly, our RNA-Seq analysis also revealed reduced expression of antimicrobial peptides, that play a vital role in the humoral immune system of *P. xylostella*.

**Conclusion:** This study demonstrates that *B. thuringiensis* plays a novel role in controlling *P. xylostella*, by suppressing the immune system.

## Introduction

The diamondback moth (DBM), *Plutella xylostella* (L.), (Lepidoptera: Plutellidae), is the main pest of cruciferous crops and is the most widely distributed of all lepidopteran pests (Talekar and Shelton, 1993). The annual management cost of DBM has reached approximately US$ 4–5 billion worldwide (Tabashnik et al., 1990; Zalucki et al., 2012). Despite the availability of modern integrated pest management approaches (Furlong et al., 2008; Grzywacz et al., 2010), most of the *Brassica* crops are treated prophylactically with insecticides (Grzywacz et al., 2010). However, the extensive use of broad-spectrum insecticides against DBM promotes the selection of insecticide resistance (Li et al., 2012; Mohan and Gujar, 2003), destroys natural enemies (Furlong et al., 2004), and pollutes the environment (Shakeel et al., 2017a). To reduce the harmful effects of insecticides, alternative control strategies have been suggested (Liu et al., 2001; Sutherland et al., 2002; Rubilar et al., 2007; Hussain et al., 2009; Diez et al., 2012), including biopesticides like *Bacillus thuringiensis*.

*B. thuringiensis*, a spore-forming Gram-positive bacterium, present in soil, leaf litter, and the microflora on the surface of leaves, is widespread in nature (Aptosoglou et al., 1997). *B. thuringiensis* produces many kinds of insecticidal crystal proteins, including proteins which are toxic to lepidopterans, and are encoded by crystal (*cry*) and cytolytic (*cyt*) genes (Crickmore et al., 1998). Cry proteins have not only been used in formulated sprays but also expressed in transgenic plants to protect them from insect attacks (Shelton et al., 2002). Cry toxins, unlike most chemical insecticides, have a distinct mode of action that involves toxin solubilization, proteolytic activation in the midgut of the insect, and binding to larval midgut proteins. In the pore formation model, toxin binding results in the formation of pores in membranes and the lysis of cells in the midgut ultimately, resulting in the death of the insect (Pigott and Ellar, 2007). Until now, insects have developed several resistance mechanisms to *B. thuringiensis* toxins, including the modification of the receptor site, alteration of binding ability and proteolysis of protoxin and/or toxin, and an elevated immune status (Pardo-Lopez et al., 2012; Zhu et al., 2016).

Like many insects, *P. xylostella* opposes microbial invaders by mounting well-adjusted immune responses. Insects, unlike their mammalian counterparts, solely rely on innate immunity, which is divided into cellular and humoral immune responses (Hoffmann, 2003). The cellular innate immune response is mediated by strong phagocytic activities of plasmatocytes while melanin synthesis, clotting, and the production of antimicrobial peptides mediated by fat body are collectively known as humoral innate immunity (Hoffmann, 2003). Although our knowledge of insect-pathogen interaction (fungi as a pathogen) has increased in recent years (Shakeel et al., 2017c; Xu et al., 2017), there are only a few reports on the interaction between *B. thuringiensis* and insects (Grizanova et al., 2014; Contreras et al., 2015); for example, when *Galleria mellonella* was infected by the oral administration of *B. thuringiensis*, an elevated immune response was observed, indicating an increased immune resistance to *B. thuringiensis* in *G. mellonella* (Grizanova et al., 2014).

Keeping the importance of insect-pathogen interaction in mind to understand the innate immune response of *P. xylostella* to *B. thuringiensis*, we investigated whether *B. thuringiensis* has the ability to suppress the immune system of *P. xylostella*. To address this question, fourth instar larvae of *P. xylostella* were fed *B. thuringiensis* at five different time points (6 h, 12 h, 18 h, 24 h, and 36 h) with a control using high-throughput Illumina sequencing and real-time quantitative PCR (RT-qPCR) techniques at the genomic level. Our findings reveal that *B. thuringiensis* has the ability to overcome the immune defense mechanism mounted by *P. xylostella* by suppressing the humoral immune system.

## Materials and Methods

### Insect Rearing and *B. thuringiensis* Preparation

A susceptible population of *P. xylostella* was obtained from the Engineering Research Center of Biological Control, Ministry of Education, South China Agricultural University, China and kept in an insecticide-free environment for 10 generations. Adults were fed 10% honey solution, and larvae were reared on Chinese broccoli. All populations were maintained at 25 ± 1°C under a photoperiod of 16: 8 h (light: dark) and 65% relative humidity. The highly pathogenic *B. thuringiensis* HD-73 strain was kindly provided by Mr. Zhang Jie of the Institute of Plant Protection, Chinese Academy of Agricultural Sciences. Bacteria from a glycerol stock (stored at -80°C) were plated on Luria-Bertani (LB) agar and grown overnight. Later, the bacterial suspension was inoculated into fresh LB medium with a proportion of 1:100, incubated for 12 h at 30°C, and finally the bacteria were centrifuged and subsequently resuspended in phosphate buffered saline (PBS). The spores were counted using a Thoma counting chamber (0.02 mm depth) and immediately used for feeding.

### Feeding *P. xylostella* Larvae With *B. thuringiensis* and RNA Sample Preparation

The lethal concentration 50 (LC_50_) dose of bacteria was determined in a pilot experiment, in which fourth instar larvae of *P. xylostella* were selected to be fed on nine increasing doses of *B. thuringiensis* treated leaf discs (by leaf dip bioassay method). The dose that came closest to killing 50% of the larvae (1.0 × 10^8^/ml) within 36 h was then selected.

The selected LC_50_ was used for the experiments. All the larvae used for the feeding experiment were starved for 2 h prior to feeding and then exposed to *B. thuringiensis* treated leaf discs at 6 h, 12 h, 18 h, 24 h, and 36 h. Larvae treated with PBS were used as a control. Twenty larvae were used in each treatment, and the whole body of the surviving larvae that ate (as determined by observing the food bites) treated leaves were used to extract RNA. Experiments were carried out in triplicate.

The Trizol Total RNA Isolation Kit (Takara, Japan) was used to extract RNA from the whole body of *P. xylostella*, following the manufacturer’s protocol. To determine the concentration and integrity of RNA, Nanodrop (Bio-Rad, United States) and Agilent 2100 Bioanalyzer (Agilent, United States) were used.

### cDNA Library Preparation and Illumina Sequencing

A total of six libraries (6 h, 12 h, 18 h, 24 h, 36 h, and control) were created by the Illumina Gene Expression Sample Prep Kit (Illumina, San Diego, CA, United States). 10 μg of total RNA, extracted from each treatment and control, was incubated with oligo (dT) magnetic beads for the isolation of the polyadenylated RNA fraction. To synthesize first- and second-strand cDNAs, random hexamers and RNase H and DNA polymerase I were used. The double stranded cDNA was purified with magnetic beads following ligation of fragments with sequencing adaptors enriched by PCR amplification. Finally, to qualify and quantify the sample libraries, Agilent 2100 Bioanalyzer and ABI Step One Plus Real-Time PCR System were used following the sequencing on the Illumina HiSeq^TM^2000 system (Illumina, United States). Illumina sequencing was performed at the Beijing Genomics Institute (BGI-Shenzhen, China).

### Genome Mapping and Analysis of Differentially Expressed Genes

**FIGURE 1:**
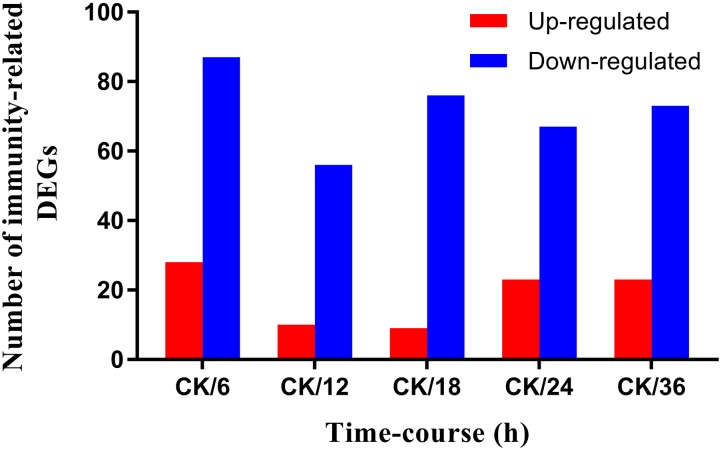
Screening of immunity-related DEGs of *Plutella xylostella* in response to *Bacillus thuringiensis* at 6 h, 12 h, 18 h, 24 h, and 36 h postinfection. CK denotes control. *Y*-axis indicates the number of differentially expressed immunity-related genes, and *X*-axis shows the ratio of pairwise comparison of all the treatments with the control.

The filtration process was carried out to remove raw reads with adapters and unknown bases >10%. After filtration, Bowtie and HISAT (Kim et al., 2015) were used to map the remaining clean reads with the reference gene and reference genome. Finally, all data were normalized as fragments per kilobase of transcript per million fragments mapped (FPKM). Differential expression analysis was carried out by a strict approach, and the threshold *p*-value was determined by using the false discovery rate (FDR) methodology for analyzing multiple tests (Kim and van de Wiel, 2008). A standard threshold (FDR <0.001 and log2 ratio ≥1) was set to identify significantly differentially expressed genes (DEGs) in the libraries. The number of differentially expressed immunity-related genes and the ratio of pairwise comparison of all the treatments with the control are represented in Figure 1.

### Identification and Hierarchical Clustering of Differentially Expressed Immunity-Related Genes

To identify *P. xylostella* immunity-related genes, the BLASTX algorithm search was compared against the Nr database using a cutoff *E*-value of 0.1. The potential candidates of *P. xylostella* immunity-related genes were confirmed by aligning the available immunity-related gene sequences to other model insect species. The reference insect species included *Danaus plexippus, Ostrinia nubilalis, Bombyx mori, Spodoptera frugiperda*, and *Manduca sexta*. Hierarchical clustering of differentially expressed immunity-related genes was performed using the pheatmap package in an R environment.

### Functional Analysis of Differentially Expressed Immunity-Related Genes

*Plutella xylostella* genome (GCA_000330985.1) was set as the background to identify significantly enriched Gene Ontology (GO) terms and Kyoto Encyclopedia of Genes and Genomes (KEGG) pathways within the DEGs dataset using a hypergeometric test and a corrected *P*-value of ≤0.05 as the threshold.

### Validation of DEG Libraries by RT-qPCR

Real-time quantitative PCR is the method of choice for analyzing the expression of genes and to confirm the results of RNA-Sequencing (RNA-Seq) (Shakeel et al., 2017b). The mRNA expression patterns of control vs. treatment groups were validated by RT-qPCR, by randomly choosing 15 immunity-related DEGs. The total RNA was isolated from the fourth instar larvae, following the same method as described earlier. First-strand cDNA (1 μg) was prepared using M-MLV reverse transcriptase (Promega, United States), following the instruction manual. RT-qPCR was carried out on a Bio-Rad iQ2 optical system (Bio-Rad) using the SsoFast EvaGreen Supermix (Bio-Rad, Hercules, CA, United States), following the instruction manual. Purity of the PCR products was confirmed by generating a dissociation curve from 65°C to 95°C (Shakeel et al., 2015) with the following PCR conditions: 95°C for 30 s, 40 cycles at 95°C for 5 s, and 55°C for 10 s. Ribosomal protein S13 (RPS13) was used as an internal control for normalization (Fu et al., 2013), and the relative expression level was calculated by the 2^-ΔΔCT^ method (Livak and Schmittgen, 2001). Each treatment included three replicates, and each reaction was run in triplicate. Primers used for RT-qPCR were designed by Primer Premier 5 (Table 1).

**Table 1:** Primers used in RT-qPCR verification for immunity-related genes.

Gene name	Forward primer (5^′^-3^′^)	Reverse primer (5^′^-3^′^)	Amplicon size (bp)
Trypsin12	CCAGCCAGCGTCCATCAATGC	ACTCGGCGTTGTTCACTGTGTATAG	146
Serpin1	TTCAGGCAAGGACTCAAGTAATAGACG	TTCTTCTACGCCATTCTTCATCAGGAC	133
PP01	CAACTATGGCTTCTCTGTGGCTCTG	GAGAACACTTGCGAGTCCAGGAAC	111
Defensin	ACAGGAGACAGTGGTTGAGGAGTC	TTGTATCTTCAGTGGCGTCTTCGTAC	98
cd-SP1	GCCAGGAGCTTCGAGAACTACAC	TTGGAGGTGCGATGCTGATGTG	84
Cecropin5	TCTGCTGCGCCTAGGTGGAAG	CGCTGGACCTGCCTTGATGATG	87
PPO5	GATGATGGTGGTGAAGATGGTGACTAC	TCCGATCAGAGCCAGACGAAGAC	106
PGRP1	GTTCATCTCAGTAACGCAACACATCAC	AGACAGCAGGCGACCAGGAG	150
SP23	GGCTCGCTACCAGAACATCAGAATG	CCACGATGAGACTCCAATGACCAC	145
Integrin7	AGTGCGACGGACTCAAGGTAGG	GAACTGCTGCCGCCACTCAC	98
βGBP8	CATCTCAGTCAGCACGGCATCAG	CGGCTCATCAGGCATCATAATCTCC	130
Lysozyme	AGTTGATAACTGACGACATCACGAAGG	CCATCCATACCAGGCGTTGAAGC	86
C-Jun2	AGCCTACCTTCTATGACGAGCAGTAC	AAGTCCAGGTCCAGCGTGAGG	82
Catalase2	TCAGAACATCACCAACAACCAGGAAG	CTTAGTGTAGAAGACGGTCGCTTGAG	184
PGRP6	CTTACGGCTACAACAGGAAGTCTATCG	TCTCACTCCACACTTCAGCAATGC	116
RPS13	TCAGGCTTATTCTCGTCG	GCTGTGCTGGATTCGTAC	100

## Results

### Summary of Illumina Sequencing and Mapping to Reference Genome

A total of 11,930,289, 12,243,539, 12,207,944, 11,905,098, 12,375,994, and 11,709,506 clean reads were generated, after filtering out adapter sequences and low-quality reads (tags with the unknown nucleotide “N”), from the six libraries (6 h, 12 h, 18 h, 24 h, 36 h, and the control). Among the data of the six clean read libraries, 75.36% to 78.22% of the clean reads were successfully mapped to the reference genome (Table 2).

**Table 2:** DGE sequencing statistics.

Sample	Clean reads	Total mapped clean reads (%)
6 h	11,930,289	75.36
12 h	12,243,539	77.79
18 h	12,207,944	78.12
24 h	11,905,098	77.68
36 h	12,375,994	77.11
Control	11,709,506	78.22

### Dynamics of *B. thuringiensis*-Responsive Immunity-Related DEGs

**FIGURE 2:**
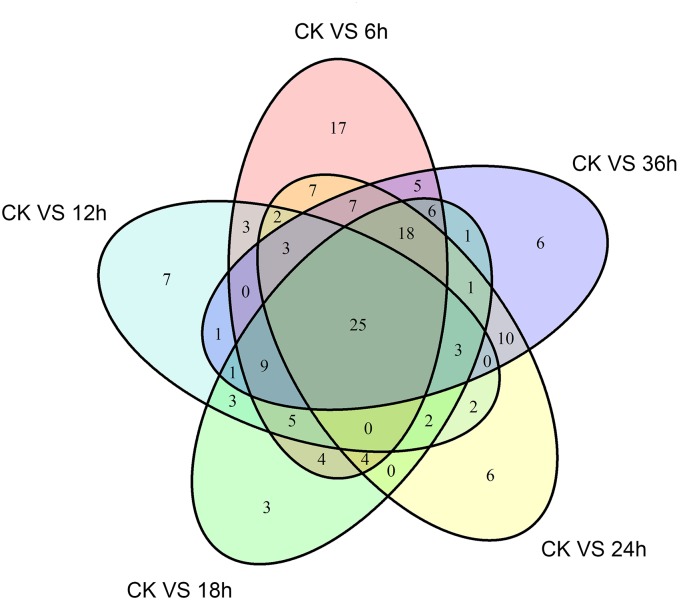
A Venn diagram of immunity-related differentially expressed genes (DEGs) in *P. xylostella* at 6 h, 12 h, 18 h, 24 h, and 36 h postinfection. The numbers in each circle show immunity-related differentially expressed genes in each comparison treatment, and the overlapping regions display genes that are commonly expressed among the comparison treatments.

A differential gene expression analysis was carried out to identify the variations in gene expression patterns between the control (PBS-treatment) and the treated groups (*B. thuringiensis*-infected) at different time points (6 h, 12 h, 18 h, 24 h, and 36 h). Our results showed that, compared to the control, there were 115 [28 (24.35%) upregulated and 87 (75.65%) downregulated], 66 [10 (15.15%) upregulated and 56 (84.85%) downregulated], 85 [9 (10.59%) upregulated and 76 (89.41%) downregulated], 90 [23 (25.56%) upregulated and 67 (74.44%) downregulated], and 96 [23 (23.96%) upregulated and 73 (76.04%) downregulated] immunity-related genes that were significantly altered in *P. xylostella* after 6 h, 12 h, 18 h, 24 h, and 36 h, respectively (Figure 1). A Venn diagram analysis showed that only 25 genes of these immunity-related DEGs were commonly expressed among all the treatments, whereas 17, 7, 3, 6, and 6 immunity-related genes were specifically expressed at 6 h, 12 h, 18 h, 24 h, and 36 h, respectively (Figure 2).

### Identification and Functional Analysis of *B. thuringiensis*-Responsive Immunity-Related Genes

**FIGURE 3:**
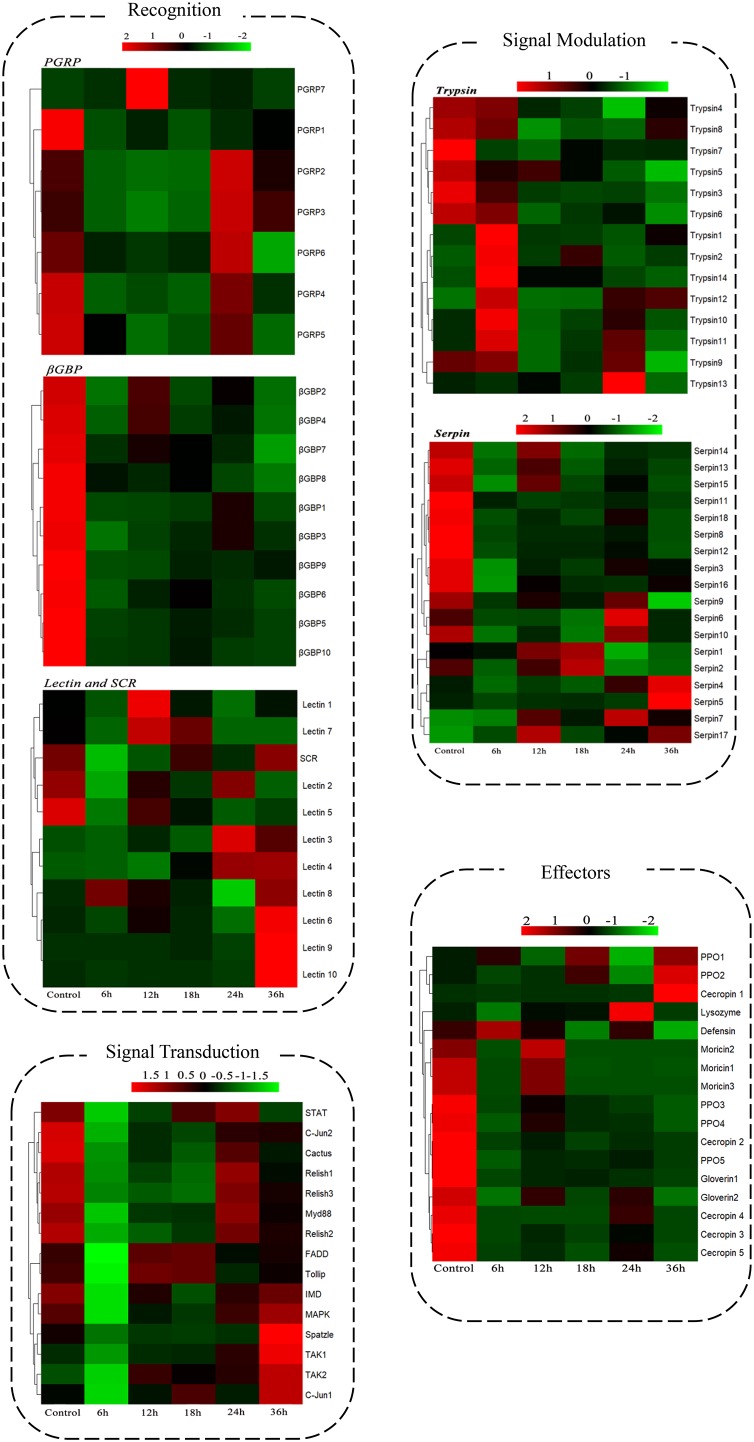
Expression patterns of the immunity-related genes of *P. xylostella* expressed in response to *B. thuringiensis* challenge. Cluster analysis expression profiles of immunity-related genes data are organized into four groups: recognition, signal modulation, signal transduction, and effectors. Six datasets were included: 6 h, 12 h, 18 h, 24 h, and 36 h postinfection with *B. thuringiensis* and one control (6 h after PBS treatment). Gene families and functional pathways (Toll, IMD) are categorized within the group. Gene names are shown on the right side, Gene ID and details are shown in the Supplementary Table S1.

A comprehensive analysis was carried out to identify *B. thuringiensis*-responsive immunity-related genes in *P. xylostella* by BLAST searches against the non-redundant sequence database and by combining GO and KEGG annotation results. To get more reliable results, genes annotated as hypothetical or unknown proteins were filtered out. Finally, the identified immunity-related genes (167) were classified into different groups, including signal recognition, signal modulation, signal transduction, effectors, and other immune molecules (Figure 3 and Supplementary Table S1).

**FIGURE 4:**
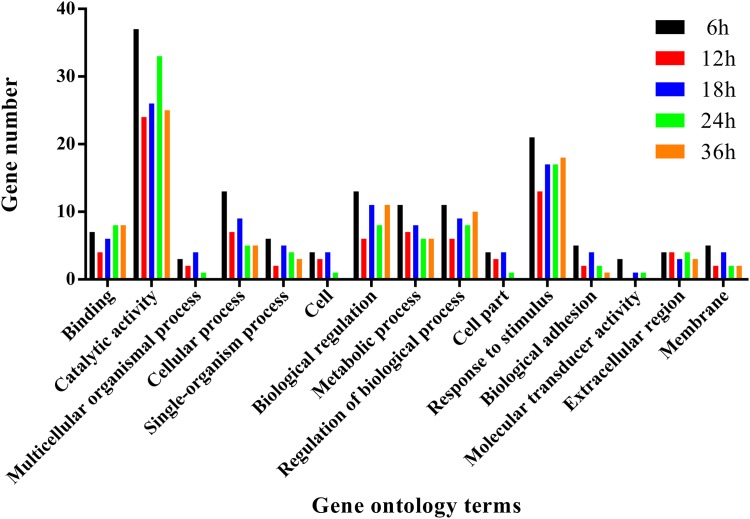
Summary of gene ontology annotations. Functional classification of immunity-related DEGs in *P. xylostella* at 6 h, 12 h, 18 h, 24 h, and 36 h postinfection using gene ontology terms.

**FIGURE 5:**
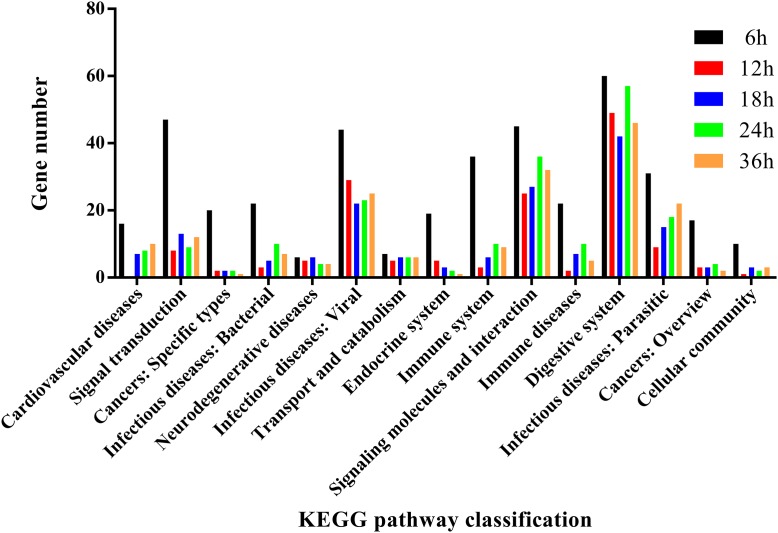
KEGG pathway annotation classification of immunity-related genes in *P. xylostella* fed with *B. thuringiensis* at 6 h, 12 h, 18 h, 24 h, and 36 h. The abscissa is the KEGG classification, and the ordinate left is the gene number.

To further analyze the functions of all significantly differentially expressed immunity-related genes in their corresponding pathways, GO enrichment and KEGG pathway analyses were performed. Our results showed that catalytic activity (32.37%) was the most enriched term following response to stimulus (19.12%), biological regulation (10.74%), regulation of biological process (9.71%), and metabolic process (8.50%) (Figure 4), whereas the digestive system (57.42%), signal molecules and interaction (36.42%), viral infectious diseases (31.94%), parasitic infectious diseases (20.23%), and signal transduction (18.16%) were identified as highly enriched categories by the KEGG pathway enrichment analysis (Figure 5).

#### Genes Involved in Microbial Recognition

In the signal recognition group of this study, most of the peptidoglycan recognition proteins (PGRPs), β-glucan-binding proteins (β-GBPs), and scavenger receptors were downregulated in response to *B. thuringiensis*; for example, PGRP4 (px-105386207), β-GBP2 (px-105389999), and β-GBP10 (px-105380182) showed a downregulated expression of 9.90-fold, 9.17-fold, and 10.01-fold, 6 h postinfection. However, a few members of the lectin family exhibited upregulated expression at 36 h with lectin6 (px-105383612), lectin9 (px-105392416), and lectin10 (px-105398492) upregulated by 3.97-fold, 12.69-fold, and 18.99-fold, respectively, postinfection.

#### Genes Involved in Signal Modulation

In this study, 67 serine protease genes showed a significant difference in expression in response to *B. thuringiensis* in the signal modulation group, including 45 serine proteases, 2 clip-domain serine proteinases, 14 trypsin-like serine proteinases, and 6 chymotrypsin-like serine proteinases. Among these serine proteases, a mixed response of upregulation and downregulation in expression after *B. thuringiensis* infection was observed at all time courses.

In this study, 18 serine protease inhibitors (serpins) were identified, and most of them were downregulated after *B. thuringiensis* infection at different time courses with serpin18 (px_105383822) significantly downregulated by 4-fold when compared with the control; however, a few serpins like serpin7 (px_105383829) and serpin5 (px_105387001) were upregulated by 2.4-fold and 2.26-fold, respectively.

#### Genes Involved in Immune Signaling Pathways

In this study, components of Toll pathway such as Spätzle, Myd88, cactus, and toll-interacting protein showed a downregulated expression in response to *B. thuringiensis* with Spätzle showing an 8-fold downregulation at the early stage of infection 6 h postinfection, compared to the control. A similar downregulated expression pattern was observed in the components of IMD (FADD, TAK1, TAK2, and relish), JNK (C-jun1 and C-jun2), and JAK-STAT (STAT) pathways.

#### Genes Involved in Immune Effector Families

Intriguingly, among immune effectors, a significantly downregulated expression of antimicrobial peptides, such as cecropins, moricins, and gloverins, was observed after *B. thuringiensis* infection, at different time courses (Figure 3 and Supplementary Table S1). A gradual variation in the expression of gloverins was observed at different time courses with gloverin1 (px-105389810) downregulated by 4.9-fold at 6 h, 3.08-fold at 12 h, 2.8-fold at 18 h, 3.3-fold at 24 h, and 4.1-fold at 36 h. Similarly, cecropin1 and moricin2 were significantly downregulated by 10-fold and 7-fold, respectively, at 6 h postinfection of *B. thuringiensis*.

#### RT-qPCR Validation of Immunity-Related DEGs

**FIGURE 6:**
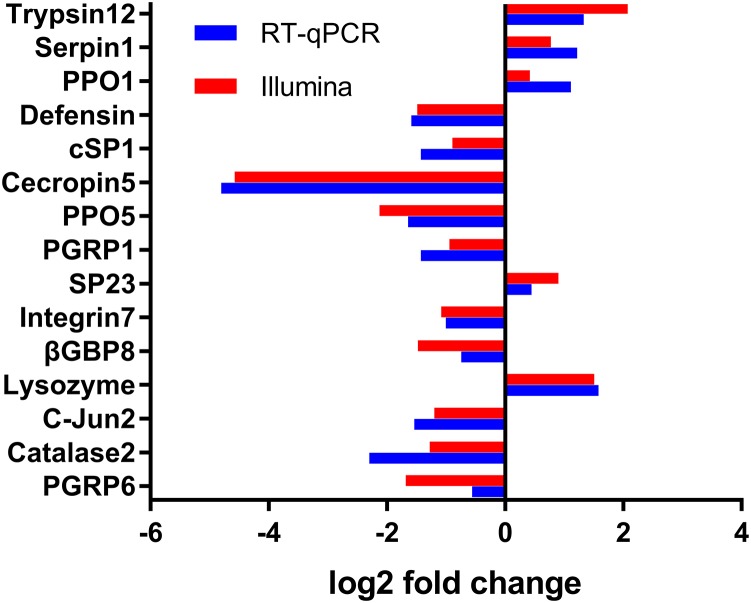
Validation of the differential expression ratio (log2) achieved by RT-qPCR and RNA-Seq for immunity-related genes. PGRP6, Peptidoglycan Recognition Protein (Px_105391041); Catalase2, Catalase (Px_105390515); c-Jun2, c-Jun(Px_105388607); Lysozyme, Lysozyme (Px_105382813); β-GBP8, Beta-1,3-glucan-binding protein (Px_105380183); Integrin7, Integrin (Px_105381998); SP23, Serine Protease (Px_105388679); PGRP1, Peptidoglycan Recognition Protein (Px_105388777); PPO5, Prophenoloxidase (Px_105393465); Cecropin5, Cecropin (Px_105394858); cd-SP1, clip domain serine protease (105395144); Defensin, Defensin (Px_105380306); PPO1, Prophenoloxidase (Px_105386870); Serpin1, Serpin (Px_105392292); Trypsin12, Trypsin-like serine protease (Px_105383571).

To verify the changes in FPKM values between different samples, expression levels of 15 DEGs were selected and verified by RT-qPCR. Consistent with the DGE sequencing data, RT-qPCR results of the 15 randomly selected genes showed a similar pattern of expression in the six libraries (Figure 6), which further confirmed that our sequencing data were reliable.

## Discussion

The use of *B. thuringiensis* based bioinsecticides is considered a promising biological alternative to control insect pests. A wide range of insecticidal proteins, with wide pathogenicity, produced by this bacterium are active against numerous pest species (van Frankenhuyzen, 2013). Presently, among the toxins produced by this bacterium, Cry toxins are the most popular biocontrol agents (Lacey et al., 2001). The pathogenesis mechanism of *B. thuringiensis* against insects is a complex process that involves several factors as well as the activation of immune responses of insects to combat infection, which is also considered as a factor contributing to tolerance against *B. thuringiensis* (Contreras et al., 2015). To better understand whether the larval immune response helps the insect to reduce the damage produced by *B. thuringiensis* (HD-73) or to overcome the immune defense mechanism mounted by the insect, we aimed to conduct a genome-wide transcriptional profiling of *P. xylostella* challenged with *B. thuringiensis* at different time courses (6 h, 12 h, 18 h, 24 h, and 36 h), using high-throughput RNA-Seq and DGE analysis.

Dynamics of immunity-related genes exhibited that most of the genes were downregulated following *B. thuringiensis* infection. Our results are in accordance with previous reports, which indicated that the number of downregulated immune related genes was higher, compared to upregulated genes in *P. xylostella* and *Bemisia tabaci* following *Isaria fumosorosea* and *Eretmocerus mundus* infection (Mahadav et al., 2008; Xu et al., 2017).

Recognition of pathogens mediated by pattern recognition molecules is the initial process of defense against intruders, eliciting the innate immune response of insects (Shakeel et al., 2017c). Until now, a variety of pattern recognition molecules have been identified, including PGRPs, β-GBPs, hemolin, scavenger receptors, and lectins (Hultmark, 2003).

In the signal recognition group of this study, most of the PGRPs, β-GBPs, and scavenger receptors were downregulated, except for a few members of the lectin family that were upregulated in response to *B. thuringiensis*. As previously demonstrated, Vip3Aa (a *B. thuringiensis* toxin) also reduced the expression of PGRPs in *Spodoptera litura*, however, contrary to our findings, an increased expression of other pattern recognition molecules was observed (Song et al., 2016). A similar trend of downregulated expression of PGRPs was observed in *P. xylostella* and *Drosophila melanogaster* following *I. fumosorosea* infection and destruxin injection (Pal et al., 2007; Xu et al., 2017). Our results suggest that PRRs like PGRPs, GNBPs, and scavenger receptors may be the target of *B. thuringiensis*, and lectins are responsible for the activation of the immune response of *P. xylostella* to *B. thuringiensis*.

Serine proteases, crucial proteolytic enzymes, play a significant role in a wide range of physiological processes, including digestion, signal transduction, and invertebrate defense responses (Ross et al., 2003). Serine proteases perform the catalytic function through the action of a catalytic triad, which is composed of His, Asp, and Ser amino acid residues (Perona and Craik, 1995). In general, serine proteases exist in the inactive pro-enzyme form and are activated by specific proteolytic cleavage (Ross et al., 2003). In our study, a mixed response of upregulated and downregulated expression of serine proteases was observed after *B. thuringiensis* infection over the time course. Notably, most of the upregulated serine proteases showed a very high expression at the initial stage of infection (6 h). A drastic variation in the gene expression after *B. thuringiensis* feeding may suggest the involvement of candidate genes in the process of protoxin activation or degradation.

Serpins are considered the most effective molecules to inactivate serine proteases when they are no longer in need. Serpins are widely reported inhibitors with documented roles in insect digestion, development, metamorphosis, and are also considered important components of the immune system (Ross et al., 2003). In this study, serpins showed downregulated expression after *B. thuringiensis* infection at different time courses. Consistent with our report, most of the serpins were downregulated when exposed to *I. fumosorosea* infection (Xu et al., 2017), however, an upregulated expression pattern of serpins was observed when *Spodoptera exigua* was challenged with the Vip3Aa toxin of *B. thuringiensis* (Bel et al., 2013).

Signal transduction pathways play a vital role in the proper functioning of the immune system in insects, as they are involved in amplifying the immune response signals and inducing antimicrobial activity (Hillyer, 2016). The signal transduction pathways include Toll, IMD, JNK, and JAK-STAT pathways. It is worth mentioning that in this study, components of all these pathways were identified, and bacterial challenges also resulted in the suppression of immune signal transduction pathways. Components of the Toll pathway such as Spätzle, Myd88, cactus, and toll-interacting protein showed a downregulated expression in response to *B. thuringiensis*. Contrary to our findings, neither the toll-like receptor gene nor the IMD gene was found to be regulated when *S. exigua* and *S. litura* were treated with the Vip3Aa toxin of *B. thuringiensis* (Bel et al., 2013; Hernández-Martínez et al., 2017).

Antimicrobial peptides, evolutionarily conserved short immunity-related proteins, play a significant role in the insect immune system by acting against a wide range of pathogens, including bacteria, viruses, fungi, or parasites (Bulet et al., 2004).

In insects, antimicrobial peptides are induced in specific tissues such as the hemocytes or body fat. In the current era, antimicrobial peptides could become a valuable alternative to conventional antibiotics, to reduce antimicrobial resistance, as they show a different mechanism of action, when compared with antibiotics (Hancock and Sahl, 2006; Vale et al., 2014).

It is worth mentioning that a significantly downregulated expression of antimicrobial peptides was observed following *B. thuringiensis* infection. Contrary to our findings, an increase in the expression of antimicrobial peptides was observed in *Trichoplusia ni* exposed to *B. thuringiensis* (Tamez-Guerra et al., 2008). However, there are a few reports of reduced expression of antimicrobial peptides in response to pathogens and parasites in *P. xylostella, D. melanogaster, Locusta migratoria, Helicoverpa armigera*, and *Meligethes aeneus* (Pal et al., 2007; Xu et al., 2017). Similar to our results, the expression of antimicrobial peptides such as lysozyme was reduced in *P. xylostella* and *L. migratoria* following fungal infection (Zhang et al., 2015; Xu et al., 2017). Moreover, the expression of antimicrobial peptides like moricin and gloverin was also suppressed in the hemocytes of *H. armigera* after *Escherichia coli* infection (Xiong et al., 2015). The suppression of immune response, especially the reduction in the expression of antimicrobial peptides in the host by entomopathogenic fungi and bacteria, in both previous reports and in this study, would have obvious benefits for the success of pathogenic fungi and bacteria. The reason for the reduced expression of antimicrobial peptides in this study might be the release of other toxins and secondary metabolites along with the main Cry1Ac toxin produced by *B. thuringiensis*, to overcome the immune system of *P. xylostella*. Thus, the ability to reduce the production of antimicrobial peptides is likely to aid fungal and bacterial survival in a variety of insect hosts.

## Conclusion

In conclusion, this study has addressed the response of the immune system of *P. xylostella* to *B. thuringiensis* exposure at different time points. *B. thuringiensis* infection led to a marked reduction in the response of the immune system of *P. xylostella* as the number of downregulated immune genes was higher at all time points, compared with upregulated genes. In the light of our findings, we speculate that *B. thuringiensis* might have released several other toxins and secondary metabolites along with the main Cry1Ac toxin, in order to overcome the immune system of *P. xylostella*. However, a series of functional validation experiments are to be performed to evaluate the immunity-related genes identified in this study.

## Author Contributions

FJ and XX conceived and designed the experiments. SL, XX, and ZZ performed the experiments. QZ, JZ, and SL analyzed the data. QZ and JX contributed reagents, materials, and analysis tools. SL, MS, and XX wrote the manuscript. MS, FJ, and XY revised the manuscript.

## Conflict of Interest Statement

The authors declare that the research was conducted in the absence of any commercial or financial relationships that could be construed as a potential conflict of interest.
